# Chicago Society of Ophthalmology

**Published:** 1884-05

**Authors:** Boerne Bettman

**Affiliations:** Secretary


### CHICAGO SOCIETY OF OPHTHALMOLOGY AND OTOLOGY.

The society met February 14, 1884. After transacting the usual routine of business, the following paper was read by Dr. E. L. Holmes: The Prognosis of Choroidal Sarcoma and Life Insurance.

"On December 3, 1874, I extirpated the right eye of Mr. ———, 47 years of age, who came to me with the following symptoms: The pupil was closed; the cornea slightly vascular; the vessels tortuous. The tension of the globe was greatly increased. The eye had been blind 12 years; had suffered from periodic pain two years, and from constant and severe pain eight months.

I expressed the opinion that the affection was glaucoma, following iritis or choroidal tumor. The case reported in Knapp's Archives, Vol. VI, proved to be a glio-sarcoma of the choroid, with small, round cells.

Within a few days I have been requested by a life insurance company to express an opinion regarding the nature of this tumor, as the patient had applied for a policy.

The prognosis in cases of choroidal sarcoma has been of great imterest to me, since my individual experience has been at variance with the opinions generally expressed on the subject.

I have been taught to believe that the average duration of life after the removal of the globe is only four years. In this case more than nine years have elapsed, with no symptoms of local nor of remote recurrence. I occasionally meet a gentleman in most excellent health,one of whose eyes I extirpated with melanotic sarcoma 14 years ago. I have enucleated the globe 26 times for this form of tumor, and have always urged the patient to inform me of any evidence of future local manifestations, or of any impairment of the general health.

In only one case have I ever received a favorable or unfavorable report. In this case there was a speedy return of melanotic growths in the orbit, in the liver, and different portions of the integument.

On the other hand, I have received subsequent reports in nearly all cases of glioma, for which I have removed the globe 24 times, and in which the glioma reappeared in the orbit within a few months.

My own experience would not lead me to regard sarcoma of the choroid as a very malignant disease. Under all the circumstances, I am surprised that I have been able to obtain the subsequent history of so few of my patients, the more so in view of my experience with glioma. The difference of opinion expressed by authors as to the ratio of fatality arises, I suspect, from the difficulty in following the subsequent history.

In reference to life insurance, I can only say I believe the application of a patient who has been affected by sarcoma of the choroid, could not be favorably entertained by the medical examiner."

Dr. Bœrne Bettman mentioned two cases of enucleation of one eye for sarcoma and glioma. In neither of these individuals had a return of the disease been noticed. Fully six years have elapsed since the sarcoma was removed, and three years since the extirpation of the retinal growth. Dr. W. Montgomery related an instance of reappearance of the tumor and death of the patient a year after enucleation of the eye. The sarcoma was in an advanced stage, and had broken through the globe.

Dr. S. Bishop then addressed the society on Acute Suppurative Inflammation of the Middle Ear—its causes and Treatment. The causes of this disease may be arranged under the three following heads: I. Natural causes. II. Traumatic causes. III. Predisposing causes. Under the first category, cold winds and drafts occupy a prominent position. Sudden and extreme changes in the temperature and humidity of the air are prolific causes of this disease. Water is a source of great evil, swimmers and sailors are frequent subjects to inflammatory attacks of the ears. The water may enter the middle ear through the Eustachian tube, form a douche, and set up a violent inflammation.

II. The second class of causes includes fracture of the temporal bone, contusions of the cranium, and sudden forcible condensation of air in the external auditory canal from any cause, such as explosions of fire-arms, and falls upon the ear.The harmful practice indulged in by parents and teachers of boxing and cuffing the ears is to be condemned. Several cases were cited of destructive inflammation and loss of hearing, caused by this obnoxious treatment. Picking and cleansing of the ears with pins, toothpicks, introduction of pebbles, beans and insects, frequently give rise to much trouble.

III. The predisposing causes are the most numerous. All those diseases, conditions, and practices which debilitate. Whatever causes congestion of the middle ear predisposes to an inflammation, such as quinine, excessive use of alcoholic beverages, strong coffee, tea, and much tobacco smoking, diphtheria, scarlet fever, and naso-pharyngeal catarrh.

During the first stage of the disease the treatment should be palliative, constant application of cold, local depletion. Three or four leeches placed in front of the tragus will afford speedy relief. The hypodermic injection of morphine is preferable to instillation of Magendie's solution in the ear. The solution cools rapidly, and the change of temperature is apt to increase the inflammation. Tobacco smoke blown into the external ear, through a pallet of cotton, is a very efficient remedy.

General refrigerant remedies must be employed; laxatives, sudorifics, diuretics, should be prescribed on general principles ; nutritous but non-stimulating remedies should be enjoined. A simple method to evacuate the middle ear is the following one. It is the reverse of the Valsalvian method of inflation. The patient is instructed to incline the head forwards, compress both nostrils with the thumb and finger, and close the mouth. Then he is directed to draw in the breath, as it were, thus producing by the inspiration a partial vacuum in the nasal cavities and pharynx. Immediately the fluid secretions in the tympanum are forced into the throat and expectorated. If there is a great swelling of the walls of the Eustachian tube, the method may not at first succeed, but is easily practiced when the tumefaction is reduced. Patients should be directed to swallow after trying this experiment, the air reënters the middle ear, and equilibrium of pressure is restored. Catheterization is recommended when this method fails. When rupture of the M. T. is inevitable, paracentesis is resorted to. During the second stage cleanliness should be strictly observed. When pain has ceased, the dry method of treatment with pulverized boracic acid or iodoform is introduced.

The carbolic acid treatment has not been satisfactory in the doctor's hands. A five per cent. solution is necessary to destroy bacteria. He is fearful that a solution of this strength might cause suppuration. One part of bromine to 1,500 parts of water, and one part of corrosive sublimate to 20,500 of water, will destroy the bacteria in 10 minutes. When the trouble shows a tendency to become chronic, the following formula is prescribed:

℞ Iodoform........................................℈i

Zinc sulph...........................................gr. ii

Aq. dist..............................................℥i

M.

Dr. Gradle opened the discussion by stating that micro-organisms are one of the causes of suppuration of the middle ear. Experiments have proven that progressive suppuration is always due to micro-organisms. As long as the discharge is profuse, bacteria are always present. They diminish and no longer increase in the discharge as the inflammation subsides. Such micrococci have been found in abscesses, and are of considerable significance. His experience in the treatment of this disease has been limited entirely to the antiseptic method. He no longer uses boracic acid, having found a more efficient substitute in the sublimate and bismuth. It checks suppuration more rapidly, and destroys the factor after the first application. A five per cent. solution of carbolic acid will not, according to the doctor's opinion, produce suppuration.

Dr. Starkey has seen good results follow the use of peroxide of hydrogen.

Dr. Holmes disapproved of paracentesis of the M. T. in acute suppuration of the middle ear. He thought the trouble would only be aggravated by such interference.

Dr. Montgomery stated that Politzer has abandoned operative treatment in acute suppuration, but practices the method in catarrhal inflammation where there is a serous secretion.

Dr. Hotz expressed doubt as to the possibility of performing the negative Valsalvian method in this disease, during the stage of inflammation, owing to the swollen condition of the mucous membrane lining the Eustachian tube. He could not satisfy himself that leeches put on the mastoid process were not as efficacious as when placed on the tragus. They relieve the pain, and should be prescribed in every case. He is fully satisfied with the action of boracic acid, and does not approve of bismuth. This powder does not dissolve, and by accumulating in the ear, might act as an irritant.

An interesting case of foreign body embedded in the fundus oculi was then shown by Dr. Hotz.

The patient was injured six months ago; a piece of steel punch penetrated the eye after passing through the upper lid. Some time afterwards he noticed a dimness of vision, followed by almost complete loss of sight. Externally, no scar or sign of inflammation is visible; the pupil is somewhat dilated; the vitreous in the upper anterior part shows some cobweb-like opacities. In the upper and inner part of the fundus, close to the papilla, is seen a black, slender, and round body, apparently about one-fourth inch ong. One end is fastened in the tissues of the eye, the other end of the splinter projects into the vitreous, reaching close up to the lens. The piece of steel is firmly fixed; it does not change its position during movements of the eye. The papilla, in fact entire fundus, is blurred, owing to the neuro-retinitis.

BOERNE BETTMAN, M.D.,
Secretary.

